# Protocol for: Sheffield Obesity Trial (SHOT): A randomised controlled trial of exercise therapy and mental health outcomes in obese adolescents [ISRCNT83888112]

**DOI:** 10.1186/1471-2458-5-113

**Published:** 2005-10-31

**Authors:** Amanda J Daley, Robert J Copeland, Neil P Wright, Jerry KH Wales

**Affiliations:** 1 The Department of General Practice and Primary Care, The Medical School, Clinical Sciences Building, University of Birmingham, UK, B15 2TT; 2 The Centre for Sport and Exercise Science, Sheffield Hallam University, Sheffield, S10 2BP, UK; 3 Sheffield Children's NHS Trust, Sheffield, S10 2TH, UK; 4 Academic Unit of Child Health, The Children's Hospital, Sheffield, S10 2TH, UK

## Abstract

**Background:**

While obesity is known to have many physiological consequences, the psychopathology of this condition has not featured prominently in the literature. Cross-sectional studies have indicated that obese children have increased odds of experiencing poor quality of life and mental health. However, very limited trial evidence has examined the efficacy of exercise therapy for enhancing mental health outcomes in obese children, and the Sheffield Obesity Trial (SHOT) will provide evidence of the efficacy of supervised exercise therapy in obese young people aged 11–16 years versus usual care and an attention-control intervention.

**Method/design:**

SHOT is a randomised controlled trial where obese young people are randomised to receive; (1) exercise therapy, (2) attention-control intervention (involving body-conditioning exercises and games that do not involve aerobic activity), or (3) usual care. The exercise therapy and attention-control sessions will take place three times per week for eight weeks and a six-week home programme will follow this. Ninety adolescents aged between 11–16 years referred from a children's hospital for evaluation of obesity or via community advertisements will need to complete the study. Participants will be recruited according to the following criteria: (1) clinically obese and aged 11–16 years (Body Mass Index Centile > 98^th ^UK standard) (2) no medical condition that would restrict ability to be active three times per week for eight weeks and (3) not diagnosed with insulin dependent diabetes or receiving oral steroids. Assessments of outcomes will take place at baseline, as well as four (intervention midpoint) and eight weeks (end of intervention) from baseline. Participants will be reassessed on outcome measures five and seven months from baseline. The primary endpoint is physical self-perceptions. Secondary outcomes include physical activity, self-perceptions, depression, affect, aerobic fitness and BMI.

## Background

The prevalence of obesity has reached alarming levels in Britain with several studies [1] reporting that the number of young people who are overweight and obese has increased notably over the past decade. This dramatic increase in overweight has not been confined to British children and adolescents; pediatric overweight is also increasing in other western countries [2,3]. While obesity is known to have many physiological consequences, the psychopathology of this condition has not featured prominently in the literature. Overweight children have increased odds of experiencing poor health related quality of life, particularly in the domains of psychosocial health, self-esteem and physical functioning [4]. Severely obese children and adolescents have been reported to have lower health related quality of life than children and adolescents who are healthy and to experience a similar quality of life as those diagnosed with cancer [5]. Cross sectional data has also demonstrated a relationship between depressive symptoms and body mass index (BMI) scores in pre-adolescent girls [6]. Overweight adolescents are more likely to be socially isolated than their normal weight counterparts [7]. Of more concern however is the evidence [8] that teasing about weight body has been consistently associated with high depressive symptoms and thoughts about attempting suicide in school children. Collectively, these findings have created a need for clinicians and researchers to address issues that are related to the long-term well-being of clinically obese young people. Without intervention, many of these negative feelings can persist into adulthood and further diminish quality of life and psychosocial health. The psychological needs of obese adolescents are unlikely to be met fully by clinicians or health professionals and exercise is not typically part of most rehabilitation programmes for obese young people. Evidently, it is important that interventions be offered to obese adolescents as part of their rehabilitation process so that they are able to participate in society to the same extent as their non-obese peers.

The health benefits of a physically active lifestyle are well documented [9] and body weight has been found to be associated with increased risk of hyperlipidemia, hypertension, insulin resistance and diabetes in later life [10]. Furthermore, physical activity for young people can contribute to the enhancement of psychological and social well-being [11]. Indeed, the value of such activities should not be underestimated given that research has consistently demonstrated that involvement in physical activity and exercise can positively improve the mental and social health of young people [12-16]. While the use of exercise as an intervention to promote psychological health outcomes has not been extensively investigated using RCT methodologies with obese children, some preliminary evidence [17,18] has indicated that participation in weight loss camps that involve physical activity can influence these outcomes. Such studies are few in number however, and typically have been poorly controlled. Where studies [19] have included psychological variables, these have not been designated as primary outcomes measures. A significant number of studies [19-23] have been based around weight loss programmes, such as restricting calorific intake and low fat diets, which, in themselves, might be a sources of distress in young people who are obese. Furthermore, although few studies have included waiting-list control groups [22], to our knowledge no published randomised controlled trial (RCT) has included an equal contact attention-control in an attempt to account for any attention effects that might be associated with different types of lifestyle or behavioural change interventions in this population. Research is also lacking on ways to tailor interventions to the needs and interests of clinically obese young people, which is perhaps partly attributable to the lack of detailed information provided by previous authors concerning their intervention approaches.

Interventions that address both the physical and psychological concerns associated with obesity are warranted. Using a randomised attention controlled methodology the Sheffield Obesity Trial (SHOT) was designed to evaluate the efficacy of exercise therapy as an intervention for improving both mental and physical health outcomes in obese young people. The primary trial hypothesis was that the exercise therapy intervention would lead to changes in participants' mental health and physical activity behaviour. By implication, these changes in physical activity behaviour might also translate into reductions in participants' BMI scores at follow-up.

## Methods/design

### Study aims

The primary aim of SHOT is to examine the effects of a supervised exercise therapy intervention in young people who are obese.

### Study design and setting

SHOT is a pragmatic randomised controlled trial where obese young people aged 11–16 years are randomised to receive exercise therapy, usual care or an attention-control intervention. The study sample will consist of adolescents who have been referred to a children's hospital in the United Kingdom (UK) for evaluation of obesity or via community and media advertisements publicising the study. Participants recruited by community adverts will have their medical eligibility to enter the trial confirmed by one of the study paediatricians (third and fourth authors). In order to facilitate recruitment and retention a £25 sport store voucher will be given to participants at the end of the intervention phase and a contribution of £2.50 towards travel expenses will be made per visit.

### Ethical considerations

Full ethical approval for this study has been obtained from the South Sheffield Local Research Ethics Committee. Written informed consent from all participants and their parents will be sought prior to their enrolment into the study. Participants will be asked to attend the dedicated project exercise facility for a familiarisation session with their parents before entering the trial

### Study interventions

**Figure 1:**
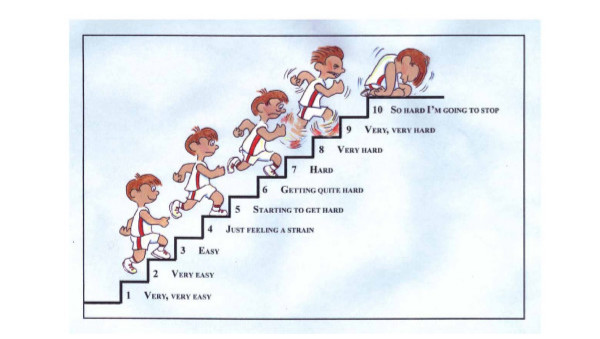
The Pictorial Children's Effort Rating Table (PCERT).

The exercise therapy sessions will take place in a dedicated project exercise therapy room housed at an English University. All exercise therapy sessions will take place one-to-one with the second author and last approximately 1 hr. Participants will be offered a range of aerobic exercise modalities, such as stepping, cycling, seated rowing, the dance mat and walking, and asked to exercise intermittently for 30 minutes, three times per week for eight weeks. The intermittent exercise will consist of a 4 minute warm up followed by four 4 minute bouts of moderate intensity exercise at 40–59% of heart rate reserve (%HRR) with 2 minute rests between each bout and 4 minute warm down. Mini games will also be included, primarily designed with fun in mind; they will also provide participants with the opportunity to experience personal development throughout the programme and introduce a small self-referenced competitive element into the sessions. Heart rate will be measured during the last minute of each 4 minute bout of exercise. Once participants have completed the eight week exercise intervention they will be given an individualised (moderate intensity) home exercise programme to follow for a further six weeks. It is hoped that the follow-up phase will help participants to move towards becoming autonomous exercisers and empower them to continue to commit to a lifestyle that involves regular aerobic exercise. During the exercise therapy sessions participants' rating of perceived exertion will be measured using the Pictorial Children's Effort Rating Table (PCERT) [24,25]. This instrument uses pictures as well as descriptive language, and has been found to reflect the changing physiological demands of given exercise tasks; higher ratings as measured by the PCERT corresponded with increases in exercise intensity [25]. Participants will be asked to estimate the exertion they feel on a 10-point scale as illustrated in Figure 1.

Motivating obese children to exercise cannot be achieved in the same way as for children of normal weight [26]. Not only are obese children physiologically different from children of normal weight, but they also have demonstrated significant emotional differences [27]. Additionally, as obese individuals tend both to be sedentary and to have had poor experiences with exercise [28], short bouts of intermittent exercise are considered most appropriate for this population. One of the common barriers to achieving long-term exercise habits in obese young people is the duration of physical activity that is often expected of them. Moreover, this can be a daunting task for these children and such high exercise demands are unlikely to be enjoyable or sustainable; this is particularly likely to be the case when obese individuals are in the early phases of adopting a physically active lifestyle. As the primary goal of this study was mental health outcomes, and not necessarily weight loss per se, intermittent exercise was considered most likely to provide opportunities for participants to experience a sense of accomplishment. The Department of Health [29] in England has recently advocated the use of short bouts of exercise accumulated throughout the day to gain health benefits.

Exercise counselling will be an integral part of the exercise sessions. It is hoped that exercise counselling will provide participants assigned to the exercise therapy group with the necessary knowledge and the psychological skills and tools to sustain changes in their exercise behaviour. This trial will use the Transtheoretical Model (TTM) [30] as the guiding framework for the exercise counselling to promote positive exercise attitudes and experiences. In line with TTM, weeks 1–4 will focus on cognitively based intervention strategies such as cognitive reappraisal and consciousness raising. During weeks 5–8, more behaviourally based interventions will be introduced, for example, goal setting, self-monitoring and finding social support. Participants will follow a broad structured curriculum of topics over the course of the intervention. Detailed descriptions of the strategies and techniques to be used during exercise counselling are outlined in Table 1. Weight loss *per se *will not be explicitly discussed with participants and no weight loss targets will be set, although sensible eating habits will be discussed and encouraged as part of the exercise therapy intervention.

**Table 1:** Exercise Counselling Protocol

**Weeks**	**Process of change**	**Exercise Counselling framework: Examples of skills and techniques used**
**1–2 Cognitive**	Consciousness Raising	**a. Review first session:**
		• How did it feel? Was it difficult/easy?
		• Did you enjoy it?
	Dramatic Relief	• Importance of exercise, why do we need to warm up & cool down
	Decisional Balance	• Heart rate monitoring, what to wear, what & when to drink
		• What to expect in the coming weeks.
		• Any questions
		
		**b. Healthy eating**
		• What is it?
		• When should I eat?
		• What type of foods are good/not so good?
		• Hand out standard dietary information sheet
		
		**c. Benefits of exercise**
		• How often?
		• How hard?
		• Where and when?

**3–4 Cognitive**	Self Re-evaluation	**d. Which physical exercises do I prefer?**
	Decisional Balance	• Previous exercise experiences, why this worked / failed.
		• What other exercises might you like to try?
		
	Consciousness Raising	**e. Do you know?**
		• Benefits of exercise
		• Importance of healthy eating
		
		**f. Are you enjoying the sessions?**
		• What do you like?
		• What do you dislike?
		• What would you change?
		• Is it what you had expected?
		
		**g. Active and healthy living**
		• Food groups, choices, portion sizes.
		• The breakfast challenge
		• Review healthy eating card.

**5–6 Cognitive and Behavioural**	Self Re-evaluation	**h. Evaluate sessions so far**
		• How do you feel about exercise now?
	Goal setting/Self-regulation	• How comfortable do you feel exercising?
		• Which exercises do you enjoy the most?
		
	Social Support	**i. Introduce goal setting**
		• What is it?
		• How might it help?
		• Set one exercise goal and one healthy eating goal for the week
		
		**j. Findings support for exercise**
		• Thinking of others who might encourage participation in exercise
		• Finding someone to talk to when exercising is difficult
		• Consider ways in which to exercise with other people

**7–8 Behavioural**	Goal setting/Self-regulation	**k. Review goals**
		• Did you achieve them?
		• If yes then well done!
	Stimulus Control	• If not then why not? What can we do to help change this?
		
	Reinforcement Management and Self-Liberation	**l. Cues for action**
		• Thinking of tasks that might prompt participation in exercise
		
		**m. Thinking about moving on from the programme**
		• Home programme phase
		• Future exercise options
		
		**n. Looking and planning ahead. SWOT analysis**
		• What will help me to exercise in the future?
		• What will stop me?
		
		**o. What have I achieved so far**
		• Review exercise
		• Review healthy eating
		• What do I want to achieve from here?
		• Thinking positively and taking positive action
		• What has been learned

An attention-control intervention (body-conditioning) has been included in this trial in an attempt to control for any attention effects that might occur in participants assigned to the exercise group. This is particularly important in the current study because the exercise therapy group will receive one-to-one sessions with the researcher. Any attention-control condition must be relevant and meaningful, particularly when used with young people. We have tried to achieve this by presenting an alternative 'exercise' group that does not involve aerobic exercise but an apparently different type of exercise in the form of body conditioning activities. Thus, like the exercise therapy group, participants assigned to the attention-control group will attend the project exercise facility for 1 hr three times per week for eight weeks. HR will be maintained below 40% HRR. Attention-control sessions will include activities such as stretching, posture, twister, as well as static juggling and catching tasks. The format of the attention-control sessions will be similar to the exercise therapy sessions; involving four 4 minute body-conditioning activities with 2 minute rest between activities. During the remainder of the session, other sedentary activities and games such as pool, darts and table football will be included to help facilitate adherence to the intervention and to make the sessions interesting and engaging. The attention group will be given a home body-conditioning programme to follow for six weeks. The attention-control group will be asked to otherwise continue with their lifestyle as normal throughout the study and will not receive exercise counselling.

The usual care comparison group will be asked to continue with their lives as usual; they will be given the opportunity to complete exercise sessions at the centre once they had completed the study.

### Determining eligibility for the study

Participants will be recruited according to the following criteria: (1) clinically obese and aged 11–16 years (Body Mass Index Centile > 98^th ^UK standard) [31]; (2) no medical condition that would restrict ability to be active three times per week for eight weeks; and (3) not diagnosed with insulin dependent diabetes or receiving oral steroids.

### Randomisation

A researcher from an independent University will perform the randomisation procedures by allocating participants to groups according to a computer generated random list.

#### Outcome measures

##### Physical self-perceptions

Physical self-perceptions served as the primary outcome measure. The Physical Self-perception Profile (CY-PSPP) was originally developed by Fox and Corbin [32] and later adapted for use with children by Whitehead [33]. The inventory contains six 6-item subscales; Sport/Athletic Competence, Attractive Body Adequacy, Condition, Strength, Physical Self-worth. The Children and Youth Physical Self-perception Profile (CY-PSPP) assesses the extent to which young people view themselves as competent in variety of physical domains. Each is devised in a structured alternative format on a scale between 1 (low score) and 4 (high score).

##### Self perceptions

Items measuring social acceptance, scholastic competence and global self-worth are to taken from Harter's Self Perception Profile for Adolescents [34]. The social acceptance subscale assessed the degree to which the adolescent feels accepted by their peers, feels popular, has lots of friends, and feels that he/she is easy to like. The scholastic competence items tap participants' perception of their competence or ability within the school context. The global self worth subscale assesses the extent to which participants like themselves as a person and the way they are living their lives. Each subscale contains six items devised in a structured alternative format on a scale between 1 (low competence) and 4 (high competence).

##### Depression

Depression will be assessed using the Children's Depression Inventory (CDI) [35]. The CDI is a 27-item self-rated symptom-orientated scale suitable for school-aged youngsters and adolescents. For each item, the child is asked to endorse one of three statements that best describe how he or she has typically felt over the past two weeks. Each response is scored as 0 (*asymptomatic*), 1 (*somewhat symptomatic*), or 2 (*clinically symptomatic*), contributing to a total CDI score that can range from 0–54.

##### Affective responses

In the absence of any exercise specific measures of affect for use with clinical child populations, items used by Ebbeck and Weiss [36] in sports settings will be included in this study. Participants responded to two subscales that assessed positive and negative affective responses over the previous week. On a scale between 1 (not at all or very slightly) to 5 (extremely) participants are asked to indicate the degree to which a series of positive and negative adjectives described how they have felt over the previous week.

##### Physical activity

The Physical Activity Questionnaire for Adolescents [37] will be used to collect detailed information about participants' involvement in different physical activities. Specifically, participants are asked about their involvement in (1) various physical activities in their spare time, (2) physical education, (3) lunchtime physical activities, (4) extra-curricular physical activities, (5) evening physical activities and (6) weekend activities. Each physical activity component is scored on a scale between 1 (not involved) to 5 (involved 5–7 times per week.

##### Anthropometrics, flexibility and aerobic fitness

As treadmill protocols engage a larger muscle mass than cycling and peak VO_2 _scores are more likely to be limited by central rather than peripheral factors [38], the poorly fit category of the modified Balke protocol [39], will be used to assess aerobic fitness. Distance walked in miles was recorded. Height was measured to the nearest completed 0.1 cm using a wall-mounted stadiometer. Weight was measured to the nearest 0.1 kg using a balance scale. From these values BMI (weight(kg)/(height(m)^2^) will be calculated and the standard deviation Score (SDS – or Z score) derived from the UK 1990 Data [40]; a child whose BMI exceeds the 98^th ^percentile for age and sex according to UK reference data in 1990 will defined as obese for the purposes of this study. Severe obesity will be defined as a BMI SDS of >+3.5 (or an adult-equivalent BMI of >40). Participants' trunk and hamstring flexibility will be assessed using the modified Acuflex I flexibility test, which allows for variations in participants' arms and legs.

### Assessment of outcomes

**Figure 2:**
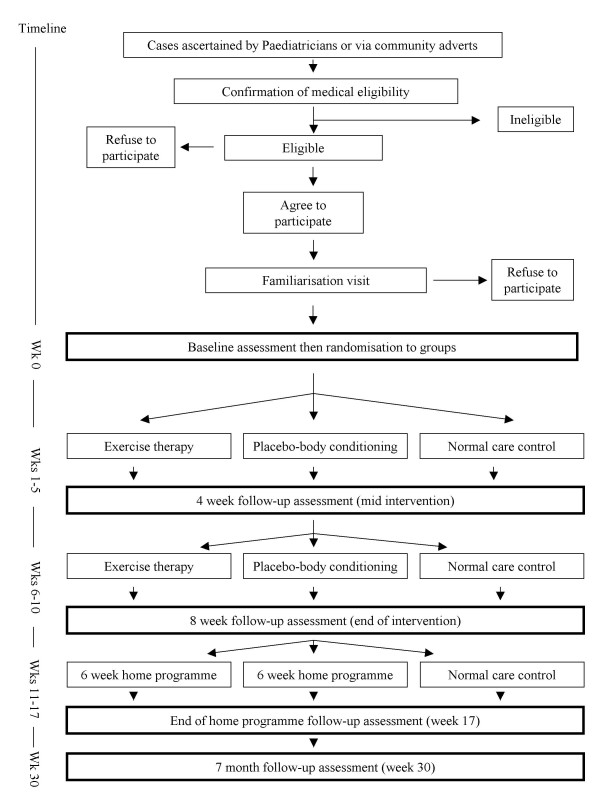
Intervention flow chart.

The main study outcomes will be assessed at baseline, as well as four (intervention midpoint) and eight weeks (end of intervention) from baseline. Participants will be reassessed on outcome measures at the end of the home intervention phase (approximately five months from baseline). To provide evidence on the possible longer-term effects of exercise therapy, a final assessment will be completed approximately seven months from baseline (see Figure 2). The second author will perform all assessments and deliver the exercise therapy and body conditioning sessions. Demographic data, including age and ethnicity and current physical activity participation will be collected at baseline. Adherence to the exercise therapy and attention-control interventions will also be monitored.

### Sample size considerations

As there has been a lack of published studies in this field power calculations have been based upon related review studies in the field of exercise and mental health. Power calculations are based upon physical self-perceptions as the primary outcome measure; (predicted effect size = 0.6, providing 80% power, p < 0.05) indicating 30 participants would be needed for each group (n = 90). A 25% dropout rate has been assumed at eight weeks from baseline indicating that 122 participants might need to be recruited.

### Statistical analysis

Differences in primary and secondary outcomes between control and intervention groups will be compared using intention to treat analysis. Imputation methods will be used to assess data losses through level drop out and loss to follow up. All results will be reported as means and 95% confidence intervals.

### Time plan for the study

Participant recruitment began in April 2002 and by January 2006 all participants will have completed the trial and follow-up assessments of outcomes.

## Competing interests

The author(s) declare that they have no competing interests.

## Authors' contributions

Amanda Daley and Jerry Wales were responsible for identifying the research question and contributing to drafting the research protocol. Robert Copeland has contributed to the development of the protocol as member of the research team. All authors were responsible for the drafting of this paper and have read and approved the final version.

## Pre-publication history

The pre-publication history for this paper can be accessed here:


